# The time frame of third molar mineralization in a northern chinese population

**DOI:** 10.1007/s12024-025-01131-3

**Published:** 2025-11-11

**Authors:** Julian Wirtz, Yu-Cheng Guo, Maximilian Timme, Sven Schmidt, Andreas Schmeling

**Affiliations:** 1 Institute of Legal Medicine, University and University Hospital Münster, Röntgenstraße 23, Münster, 48149 Germany; 2 Department of Orthodontics, Stomatological Hospital of Xi’an Jiaotong University, 98 XiWu Road, Xi’an, Shaanxi 710004 People’s Republic of China

**Keywords:** Dental age, Third molar, Mineralization, OPG, Demirjian stages

## Abstract

A key criterion in dental age determination is the assessment of third molar mineralization. The objective of this study was to provide reference data for Demirjian stages D-H of teeth 38 and 48 in a northern Chinese population. To this end, 988 digital orthopantomograms (OPGs) of northern Chinese individuals aged 12–26 years were examined. Of the total sample, 822 OPGs showed at least one evaluable lower third molar. The mean ages for both teeth in males are above 18 years from stage G onwards. In females, the mean ages for both teeth are above 18 years from stage F onwards. The youngest individuals with mineralization stage H were older than 18.0 years in both sexes. Since studies with larger case numbers have also reported minimum ages for this stage below the age of 18, the minimum ages for stage H presented in this study cannot be recommended for age assessment practice. In order to avoid stage misclassifications, which are particularly problematic when applying the minimum age principle, stage classifications in future studies should be made consensually by experienced examiners.

## Introduction

In recent years, forensic age assessment has become a research focus in legal medicine [1]. The background to this development is increasing cross-border migration, which has led to a growing proportion of the population in many countries who cannot prove their chronological age with valid identity documents. Forensic age assessment is requested by authorities and courts in order to avoid discriminating against migrants with dubious age information on the basis of age and to be able to correctly carry out procedures that are dependent on age. Important legally relevant age limits are 14 and 18 years of age [2]. Current research topics in forensic age assessment include X-ray-free imaging techniques [3–5] and the use of artificial intelligence for age assessment [6–8]. However, further research is also needed to improve established methods of forensic age assessment.

If there is a legal basis for X-ray examinations without medical indication, the international and interdisciplinary Study Group on Forensic Age Diagnostics (AGFAD) recommends combining a physical examination with anamnesis, an X-ray examination of the hand, and a dental examination with an orthopantomogram (OPG) for age assessments in adolescents and young adults. If the development of the hand skeleton is complete, an additional CT scan of the clavicles should be performed [2, 9, 10].

In dental examinations, the developmental characteristics of eruption and mineralization of third molars are of crucial importance for age assessment [11, 12]. Future studies will have to show whether degenerative dental characteristics, such as the visibility of the root pulp and periodontal ligament of third molars or the radiologically assessable Gustafson characteristics, can also be recommended for forensic age assessment [13–16].

There are several stage classifications for assessing the mineralization of third molars. The most commonly used classification is Demirjian’s classification (1973) [17]. In addition to Demirjian’s classification, Olze et al. (2005) analyzed the classifications of Gleiser and Hunt (1955), Gustafson and Koch (1974), Harris and Nortje (1984), and the classification according to Kullman et al. (1992) [18–22]. The classification developed by Demirjian et al. (1973) was found to be the most suitable for use in assessing the mineralization status of third molars [17, 18].

Depending on the legal issue, the expert report on age assessment will specify the minimum age and/or the most probable age of the person examined. If exceeding a legally relevant age limit is to be assessed with the highest degree of certainty (“with a probability beyond reasonable doubt”), the minimum age concept is applied. The minimum age is derived from the minimum age in the reference study used for the developmental stage identified. It is therefore the age of the youngest person in the reference population who exhibits the respective stage of development. When examining several age markers, the highest minimum age is decisive. The application of the minimum age concept ensures that the forensic age of the person being assessed is never stated too high, but is practically always below the actual age. This avoids unjustified legal discrimination against persons without valid identification documents.

Since there are generally no forensically usable reference studies available for the regions of origin of the persons to be examined, the question arises as to whether there are significant developmental differences between different ethnic groups that must be taken into account in age assessment practice. Olze et al. (2004) were able to show that Europeans occupy a middle position in the mineralization of third molars, while Africans show a comparatively accelerated development and Asians a comparatively delayed development [23]. The aim of this study was to present reference data for the time frame of third molar mineralization in a Chinese population.

## Materials and methods

A total of 988 digital OPGs of northern Chinese patients aged 12–26 years were available. Table 1 shows the age distribution of the sample, separated by sex. All OPGs were taken at the Department of Oral Radiology at the Affiliated Stomatological Hospital of Xi’an Jiaotong University Health Science Center, China, from 2016 to 2020.

**Table 1:** Age and sex distribution of the study population

Age (years)	Males (*n*)	Females (*n*)	Total (*n*)
12	30	34	64
13	32	34	66
14	33	32	65
15	33	33	66
16	30	32	62
17	33	49	82
18	35	35	70
19	32	32	64
20	32	37	69
21	30	30	60
22	30	32	62
23	34	30	64
24	30	32	62
25	30	31	61
26	41	30	71
Total	485	503	988

The inclusion criteria were as follows: (1) northern Chinese origin, (2) age 12–26 years, (3) free of any medical disease or surgical condition that could affect the presence and development of third molars, (4) normal development and dental conditions, (5) at least one existing lower third molar. The exclusion criteria were (1) insufficient image quality affecting third-molar visualization, (2) tilting of the third molar leading to impaired stage determination, (3) single-rooted third molar, (4) fused tooth roots.

The mineralization status of the lower third molars was assessed using the stage classification by Demirjian et al. (1973) [17]. All OPGs were assessed by three examiners. Two examiners had more than 20 years of experience in assessing third molar mineralization using the Demirjian stages. The third examiner was a doctoral student who was calibrated prior to the assessments. The calibration of the doctoral student was carried out in several stages. First, the stage classification according to Demirjian et al. (1973) was explained to the doctoral student [17]. The doctoral student then evaluated 50 OPGs that did not originate from the study group. Incorrect assessments were discussed with the doctoral student. The doctoral student then evaluated another 50 OPGs that were also not from the study group. Incorrect assessments were discussed with the doctoral student after this round as well. Where the ratings of the three examiners were found to disagree, the examiners reached a consensus through subsequent discussion of the cases.

## Results

Of the 988 OPGs available, 822 OPGs showed at least one evaluable lower third molar. Table 2 presents the age and sex distribution of the evaluable OPGs. The evaluable OPGs showed 1598 teeth. Of these, 479 teeth were not evaluable. Reasons for non-evaluability were: tilted teeth (206 teeth), insufficient image quality (203 teeth), single-rooted teeth (52 teeth) and fused tooth roots (18 teeth).

**Table 2:** Age and sex distribution of the study population with evaluable OPGs

Age (years)	Males (*n*)	Females (*n*)	Total (*n*)
12	21	23	44
13	22	27	49
14	22	23	45
15	29	28	57
16	27	29	56
17	33	45	78
18	30	31	61
19	29	26	55
20	29	34	63
21	22	28	50
22	22	28	50
23	26	23	49
24	26	30	56
25	26	24	50
26	35	24	59
Total	399	423	822

**Fig. 1:**
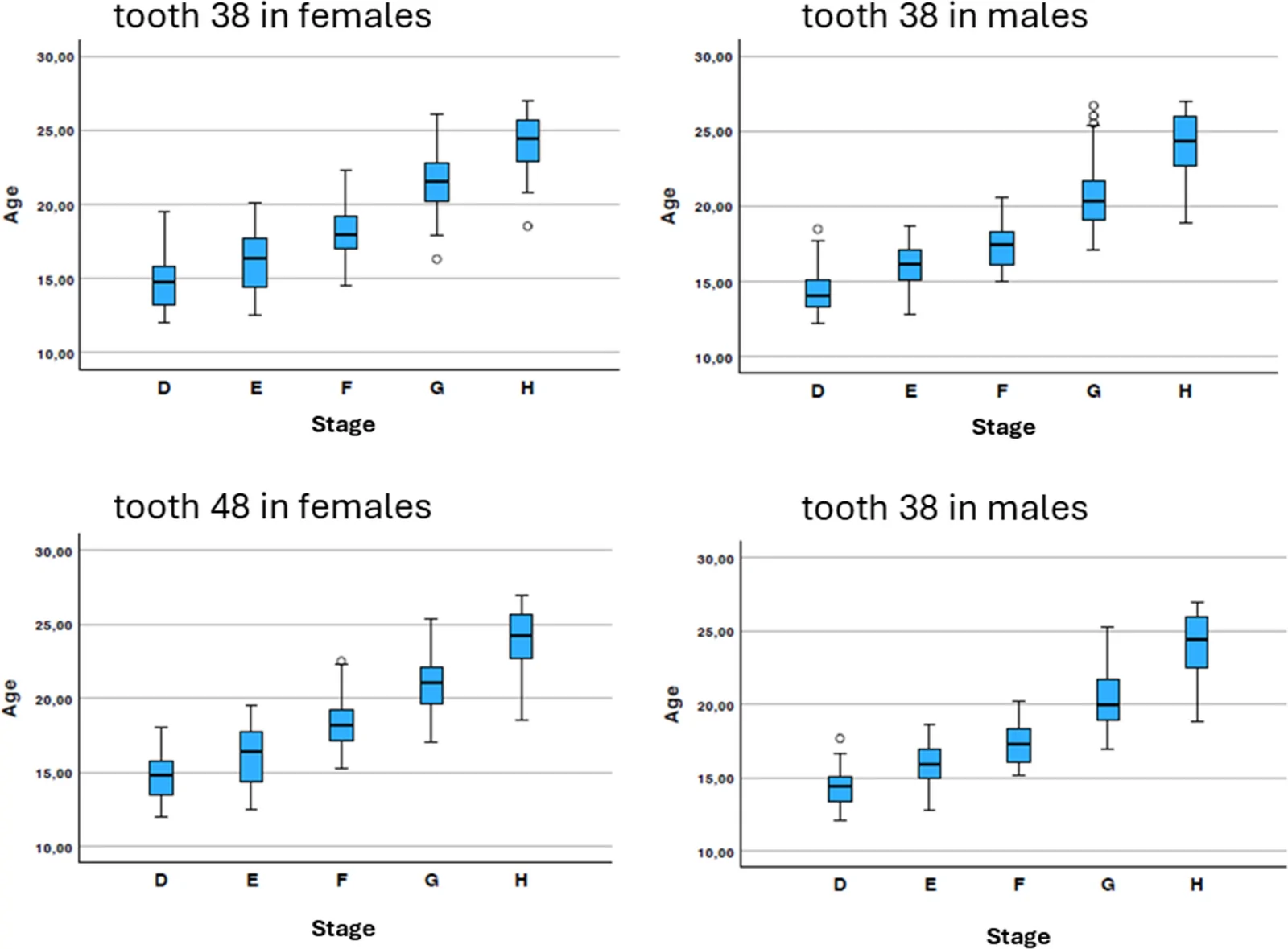
Boxplots illustrating the distribution of ages for Demirjian stages D–H of lower third molars (teeth 38 and 48), shown separately for males and females

Tables 3 and 4 detail the number of cases, minimum, maximum, mean values with standard deviations, as well as medians with lower and upper quartiles for the age of mineralization of teeth 38 and 48 according to Demirjian’s stages D-H for males and females. Due to the small number of cases, no statistical measures are presented for stages A-C. Figure 1 depicts the age distribution for Demirjian stages D–H of teeth 38 and 48, presented separately for males and females.

**Table 3:** Statistical measures for stages D to H for teeth 38 and 48 in males. Ages in years. *SD* standard deviation, *LQ* lower quartile, *UQ* upper quartile

Tooth	Stage	*N*	Min	Max	Mean	SD	Median	LQ	UQ
38	D	39	12.24	18.48	14.38	1.46	14.08	13.31	15.15
38	E	49	12.77	18.67	16.00	1.44	16.15	15.05	17.13
38	F	48	15.00	20.58	17.40	1.52	17.42	16.09	18.28
38	G	84	17.13	26.69	20.64	2.14	20.37	19.06	21.67
38	H	113	18.85	26.97	24.15	2.10	24.32	22.64	26.00
48	D	39	12.10	17.68	14.30	1.31	14.43	13.33	15.15
48	E	48	12.77	18.67	15.86	1.41	15.87	14.91	17.00
48	F	43	15.11	20.22	17.27	1.42	17.28	16.07	18.39
48	G	80	16.96	25.26	20.40	2.03	19.98	18.95	21.73
48	H	121	18.85	27.00	24.15	2.13	24.47	22.49	26.00

**Table 4:** Statistical measures for stages D to H for teeth 38 and 48 in females. Ages in years. *SD* standard deviation, *LQ* lower quartile, *UQ* upper quartile

Tooth	Stage	*N*	Min	Max	Mean	SD	Median	LQ	UQ
38	D	66	12.02	19.55	14.70	1.74	14.75	13.20	15.85
38	E	47	12.49	20.09	16.13	1.85	16.35	14.38	17.77
38	F	75	14.57	22.26	18.10	1.69	17.95	17.02	19.25
38	G	77	16.31	26.08	21.54	1.95	21.57	20.22	22.90
38	H	90	18.54	26.98	24.23	1.81	24.44	22.82	25.70
48	D	62	12.02	17.98	14.73	1.63	14.85	13.43	15.77
48	E	51	12.49	19.55	16.10	1.88	16.35	14.38	17.77
48	F	72	15.23	22.50	18.26	1.65	18.18	17.13	19.23
48	G	79	17.08	25.33	21.07	1.90	21.01	19.57	22.21
48	H	99	18.54	26.98	24.15	1.95	24.25	22.65	25.72

Both in males and females, the data reveal that the minima and maxima, as well as the mean and median values of chronological age, increase with progressive mineralization stages. The mean ages for stages D-H are greater than 14.0 years for both teeth and both sexes. The mean ages for males are above 18 years for both teeth from stage G onwards. For females, the mean ages for both teeth are above 18 years from stage F onwards. The minimum ages for both teeth and both sexes are greater than 14.0 years from stage F onwards. The minimum ages for stage H are above 18.0 years for both teeth and both sexes.

## Discussion

The aim of this study was to collect statistical measures for the temporal progression of third molar mineralization in a northern Chinese population. Tables 3 and 4 show the minima, maxima, means with standard deviations, and medians with lower and upper quartiles for mineralization stages D to H, separated by sex. When applying these measures in age assessment practice, it should be noted that the mean values and standard deviations for stage H depend on the upper age limit of the sample examined and are therefore not suitable for age assessments.

Several studies are available on third molar mineralization in Chinese populations. Zeng et al. (2010) studied 3153 OPGs of southern Chinese individuals aged 4–26 years [24]. Li et al. (2012) examined 2078 OPGs from a western Chinese population [25]. The age of the individuals was between 5 and 23 years. Qing et al. (2014) studied OPGs of 2192 southern Chinese subjects aged between 8 and 25 years [26]. Guo et al. (2015) examined 3212 OPGs from a northern Chinese population [27]. The age of the individuals was between 5 and 25 years. Liu et al. (2018) studied 2519 OPGs of central southern Chinese individuals between the ages of 8 and 23 years [28]. Table 5 shows the mean ages with standard deviations from these studies for stages D-H of tooth 48. For comparison, the values for a German and a South African population are also given. Olze et al. (2004) examined OPGs from 1,430 Germans and 584 South Africans in the 12 to 26 age group [23].

**Table 5:** Geographic origin and statistical measures (mean ± standard deviation) in years for stages D to H of tooth 48 of the present study and other studies investigating third molar mineralization. Values are rounded to one decimal place

	Present study	Zeng et al. (2010)	Li et al. (2012)	Qing et al. (2014)	Guo et al. (2015)	Liu et al. (2018)	Olze et al. (2004)	Olze et al. (2004)
Geographic origin	Northern Chinese	Southern Chinese	Western Chinese	Southwestern Chinese	Northern Chinese	Central Southern Chinese	German	South African
Stage D males	14.3 ± 1.3	13.5 ± 1.5	14.2 ± 2.2	14.4 ± 1.7	12.9 ± 1.4	13.8 ± 1.9	16.1 ± 3.0	13.9 ± 1.3
Stage D females	14.7 ± 1.6	13.9 ± 1.7	14.6 ± 2.2	14.8 ± 1.6	13.0 ± 1.6	14.1 ± 2.2	15.8 ± 2.6	14.5 ± 2.3
Stage E males	15.9 ± 1.4	15.1 ± 1.6	16.7 ± 2.4	15.7 ± 1.8	15.6 ± 2.2	16.5 ± 2.3	16.7 ± 2.1	15.2 ± 2.4
Stage E females	16.1 ± 1.9	15.8 ± 1.8	16.5 ± 2.3	16.5 ± 2.4	15.5 ± 2.3	17.0 ± 2.3	17.2 ± 2.4	15.9 ± 2.3
Stage F males	17.3 ± 1.4	17.1 ± 1.8	18.0 ± 2.1	17.4 ± 1.8	19.0 ± 2.8	17.7 ± 2.3	18.2 ± 2.1	18.7 ± 2.3
Stage F females	18.3 ± 1.6	17.8 ± 2.1	18.2 ± 2.3	17.3 ± 1.7	19.2 ± 2.8	18.6 ± 2.2	19.0 ± 2.5	17.4 ± 2.5
Stage G males	20.4 ± 2.0	19.3 ± 1.8	19.4 ± 2.3	18.4 ± 1.5	19.8 ± 2.6	20.0 ± 2.0	21.2 ± 1.9	20.8 ± 2.2
Stage G females	21.1 ± 1.9	20.6 ± 2.2	19.8 ± 1.9	19.3 ± 1.8	21.1 ± 2.5	20.1 ± 1.9	21.6 ± 2.1	19.8 ± 2.3
Stage H males	24.2 ± 2.1	22.7 ± 2.3	21.2 ± 1.7	21.9 ± 2.4	22.5 ± 2.0	21.1 ± 1.9	22.5 ± 1.7	22.6 ± 1.9
Stage H females	24.2 ± 2.0	23.4 ± 2.0	21.7 ± 1.5	21.9 ± 2.4	22.5 ± 1.9	21.1 ± 1.7	22.9 ± 1.7	22.4 ± 1.9

An analysis of the mean ages in these studies does not reveal a consistent picture. In the studies by Li et al. (2012), Guo et al. (2015), and Liu et al. (2018), the mean ages in the low and high stages are lower and in the middle stages higher than in the present study [25, 27, 28]. Noteworthy are differences of more than two years in stage H in the studies by Li (2012) and Liu et al. (2018) [25, 28]. In the study by Zeng et al. (2010), the mean ages in all stages are lower than in our study [24]. In the South African population, the mean ages are predominantly lower than in our study [23]. In the study by Quing et al. (2014), the mean ages in the lower stages tend to be higher and in the high stages tend to be lower than in our population [26]. In the German population, except for stage H, the mean ages are higher than in our study [23].

Knell et al. (2009) and Gelbrich et al. (2010) have shown that the age range of the study population influences the mean ages if the youngest and oldest individuals who can exhibit the respective stages are not included [29, 30]. Truncation of the upper age segment leads to a reduction in the mean ages, while truncation of the lower age segment leads to an increase in the mean ages. This effect may partly explain the differences in mean ages in the studies compared. In our study population, the youngest individuals were 12 years old. In the study groups of Zeng et al. (2010), Li et al. (2012), Quing et al. (2014), Guo et al. (2015), and Liu et al. (2018), the youngest individuals were between 4 and 8 years old [24–28]. Since stage D can occur before the age of 12 [24, 27, 28], the lower mean ages for stage D in the studies by Zeng et al. (2010), Li et al. (2012), Guo et al. (2015) and Liu et al. (2018) can be explained by the lower age limits of their samples [24, 25, 27, 28]. However, the mean ages for stage D in the study by Qing et al. (2014), which also has a lower age limit than our sample, are slightly higher than in our study [26]. The fact that the mean ages for stages G and H in our study are significantly higher than in almost all other studies compared cannot be adequately explained by the upper age limit of the samples.

Gelbrich et al. (2010) pointed out that the mean ages depend not only on the age range of the sample, but also on the age distribution within the sample [30]. A predominance of younger subjects leads to a decrease in the mean ages, and a predominance of older subjects leads to an increase in the mean ages. This effect could be another reason for the different mean ages.

The studies compared do not allow to deduce any influence of ethnicity or different dietary habits on the mean ages. This becomes particularly clear when comparing our data with the data from Guo et al. (2015) [27]. Both studies examined a northern Chinese population from the same catchment area. However, the mean ages differ by up to 1.7 years, which cannot be explained by the influence of ethnicity or different dietary habits.

If exceeding legally relevant age limits must be proven with the highest degree of certainty, the minimum ages of the mineralization stages are decisive. For the most forensically significant age limit of 18 years, the minimum age of stage H is of interest. In our study, this minimum age was 18.8 years for males and 18.5 years for females, and thus above 18.0 years for both sexes.

Table 6 shows the minimum ages of stage H of tooth 48 in various Chinese populations. In the study by Bai et al. (2008), the minimum ages are higher, while in the other studies they are lower than in our study [31]. It can generally be assumed that the number of cases influences the minimum age. The higher the number of cases, the higher the probability that the youngest individuals in the respective stage were included in the sample. In the studies by Zeng et al. (2010), Guo et al. (2015), and Liu et al. (2018), significantly more individuals were examined in stage H than in our study [24, 27, 28]. In the studies by Guo et al. (2015) and Liu et al. (2018), the minimum ages for both sexes were below 18.0 years, while in the study by Zeng et al. (2010), the minimum age for females was below 18.0 years [24, 27, 28]. The minimum ages we have reported are therefore not suitable for age assessment practice.

**Table 6:** Geographic origin, case number (n) and minimum ages (Min) in years for stage H of tooth 48 of the present study and other studies investigating third molar mineralization. Values are rounded to one decimal place

	Present study	Bai et al. (2008)*	Zeng et al. (2010)	Guo et al. (2015)	Liu et al. (2018)
Geographic origin	Northern Chinese	Central Chinese	Southern Chinese	Northern Chinese	Central Southern Chinese
n male	121	16	218	277	173
Min male	18.8	22.0	18.1	17.6	16.5
n female	99	20	356	378	147
Min female	18.5	19.6	17.6	17.2	16.1

* not clear which lower third molar has been examined

In the study by Liu et al. (2018), the minimum ages are surprisingly low, at 16.5 years for males and 16.1 years for females [28]. In addition to differences in case numbers, incorrect stage determinations may also be a cause of differing minimum ages. These are particularly problematic when applying the minimum age principle. In most previous studies, all stage determinations were made by a single examiner. To determine intra-observer agreement, some or all of the cases were re-evaluated by the same examiner. To determine inter-observer agreement, some or all of the cases were evaluated by a second examiner. The agreement was never 100%, which means that there must have been misclassifications. The first determinations made by the first examiner were generally used to calculate the statistical measures. It can be assumed that this approach resulted in misclassifications being included in the evaluations. To minimize such misclassifications, stage determination in our study was performed by consensus.

## Conclusions

This study presents reference data for third molar mineralization of stages D-H in a northern Chinese population. A comparison of the data with studies on Chinese, German, and South African individuals does not reveal a consistent picture. Statistical artifacts are more likely to explain the population differences observed than ethnicity or dietary habits. The youngest individuals with mineralization stage H in our study were older than 18.0 years in both sexes. Since studies with larger case numbers have also reported minimum ages for this stage below 18 years of age, the minimum ages we present are not suitable for age assessment practice. In order to avoid misclassification of stages, which is particularly problematic when applying the minimum age principle, stage classifications in future studies should be made consensually by experienced examiners.

### Key points

1. This study provides reference data for third molar mineralization stages D–H in a northern Chinese population, based on 988 digital orthopantomograms.
2. In this sample the minimum age for stage H exceeded 18.0 years in both sexes; however, because larger studies have reported minimum ages <18, these study-specific minima are not recommended for standalone forensic age determination.
3. Differences between studies are more likely due to sampling and methodological effects than to ethnicity or diet, underlining the need for consensus-based staging by experienced examiners to reduce misclassification.
